# Progress in Bacteriology

**Published:** 1903-01-24

**Authors:** 


### PROGRESS IN BACTERIOLOGY.

**Bovine and Human Tuberculosis.**—Orth [1] favours the theory of the identity of these two conditions. The contradictory results obtained by previous observers he attributes to their different methods of inoculation. The most effectual method in his hands has been the introduction of portions of tuberculous kidney into the peritoneum of the animal to be inoculated. Using tubercle bacilli obtained from a human source he succeeded in exciting tubercular lesions in one out of three calves, one out of three pigs, and in all three goats experimented upon. He argues that if tubercle bacilli of human origin are thus capable of exciting lesions indistinguishable from the ordinary tuberculosis found in animals, we cannot neglect the possibility of the process being reversed and bovine tuberculosis being transmitted to human beings. Similarly Fibiger and Jensen,[2] experimenting with tubercular material obtained from human intestinal lesions, have succeeded in producing typical tubercular lesions in the calf. The cases, five in number, from which the material was obtained were all apparently primary infections of the bowel due to the ingestion of contaminated milk. Emulsions were made of the material, and this, either with or without passage through guinea-pigs, was inoculated into the peritoneum of the calf. One point of great interest was elucidated, namely, that the virulence of the bacilli varied inversely as the age of the patient, and that a long residence in the human body tended to lower the virulence of the tubercle bacillus almost to a vanishing point. A possible solution of this question of the identity of human and bovine tuberculosis may be found in the perfection of the agglutination test. If the serum of human tubercular subjects will agglutinate the cultures of bovine tubercle bacilli, and *vice versâ*, then the two diseases cannot be different. The difficulty is, however, that the agglutination reaction in tuberculosis is extremely difficult to perform owing to the lack of homogeneity in the cultures. Arloing and Courment [3] recommend that the tubercle cultures be first made on glycerinated potato medium. Fatty, shining colonies are re-inoculated into glycerinated bouillon and the tubes well shaken daily. Those cultures are then chosen in which the bacilli are most widely separated, and from these subcultures are made and are ready for use in from eight to twelve days. The dilution employed is 1 in 20, and the reaction may be observed both macroscopically or microscopically. A new differential stain for tubercle bacilli is recommended by Müller.[4] The films are fixed and stained with carbol fuchsin in the usual way. They are then washed with 70 per cent. alcohol and with water; the final decolorisation is completed in one of two ways, either by soaking for 15 minutes in a 10 per cent. solution of calcium percarbonate, or by immersing in hydrogen peroxide solution for a few minutes. The film is then counterstained with methylene blue. By this process it is claimed that the tubercle bacillus is never decolorised. It is not stated how other acid-resisting bacilli react to this staining method. Folli,[5] who examined six cases of gangrene of the lung, and found acid-resisting bacilli in three, states that these pseudo-tuberculous bacilli are longer, finer, and more pointed than the true bacillus of Koch, that they generally occur in clumps or chains, and stain uniformly throughout a brighter red without the deep granules seen in the true variety. A 5 per cent. solution of tartaric acid will decolorise the pseudo-bacillus in five minutes, whereas it takes 20 minutes to decolorise the true tubercle bacillus. Probably these acid-resisting forms should, like the tubercle bacillus itself, be classified with the actinomycetes or streptothrices. All types may in culture media or in tissues show branching and mycelial development, and if inoculated give rise to nodular growths. Abbott [6] and Gildersleave have recently suggested and brought out evidence such as the above to show that the tubercle bacillus is but a phase of the more complex parasite. With reference to the tuberculin test for tuberculosis in cattle, Adami, and Martin [7] state that without exception all of the ten animals experimented upon in their recent research, which gave the reaction were subsequently, by post-mortem examination, proved to be tuberculous. In nine the disease was generalised or extensive, and all the evidence pointed to infection having taken place by the respiratory tract. None of the ten cows examined showed tubercles in the mammary gland. The tuberculin injection should not be repeated for 30 or 40 days as a second reaction cannot be obtained so soon. Although the mammary glands were not infected, tubercle bacilli were sometimes present in the milk through which they are excreted with a diminished virulence. The greater the infected condition of the cow, the greater the chance of tubercle bacilli being found in the milk. If the cow be only slightly diseased the bacilli in the milk will have but a very feeble infectivity. Thus of 44 guinea-pigs inoculated only two succumbed, and of 42 rabbits only one became infected. Young calves fed on such milk developed no signs of tuberculosis. From time to time the number of bacilli in the milk showed rapid temporary increase. Only one out of the ten cows showed strong clinical evidence of the disease.

**Pneumococcus.**—Washbourne,[8] whose Croomian lectures have been completed and edited by Hale-White, and Eyre, suggested that this organism is best designated the streptococcus lanceolatus. As generally found it appears as a diplococcus or in short chains of three or four cocci. Groups and tetrads are rare. In cultures the organism assumes an oval shape and loses its capsule, and in the peritoneal exudation of infected animals it may assume a bacillary form. This pleomorphism makes it important that suspected cultures should be tested by animal inoculations. The best medium is blood agar slightly alkaline in reaction. Though easily killed by physical and chemical agencies, it may be kept alive for as long as 50 days on blood agar, on which it forms a canary-coloured pigment deepening to a chocolate shade. In growth it forms lactic acid. No soluble toxin is produced. Most of the smaller animals succumb readily to the pneumococcus, but pigeons and fowls are immune. Acute septicæmia is generally the cause of death in inoculated animals, but by adopting special methods the following lesions have been produced: endocarditis, otitis, conjunctivitis, and pneumonia. The very widest differences as to virulence are to be met with amongst pneumococci, and there are certainly many distinct types or varieties. Immunity can be conferred on the lower animals by intravenous injections of dead cultures of the pneumococcus, and the serum of the immune animal has a protective influence if injected into others. Most of the antipneumococcic serums prepared have had but a feeble action, the most potent being that prepared by Pane from donkeys. This protective substance is probably formed in the bone-marrow. The blood of persons suffering from pneumonia has a feeble agglutinating power for cultures of the pneumococcus. As pneumococci are normally present in the mouth, ordinary staining of sputum is not sufficient to establish as diagnosis of pneumonia. Some of the sputum should be injected under the skin of the ear of a rabbit, and if marked œdema result within 24 hours the condition is due to a virulent pneumococcus and not to the avirulent varieties normally present in the mouth. The pneumococcus has also been demonstrated in the lungs of persons apparently healthy, and in cases of pneumonia it can frequently be isolated from the blood.

**The Diphtheria Bacillus and Scarlet Fever.**—Gordon Pugh[9] classifies the diphtheria bacilli under three heads:—(1) Virulent Klebs Loeffler bacilli; (2) non-virulent Klebs Loeffler bacilli; (3) Hoffmann bacilli. To differentiate between groups 1 and 2 and group 3 the following points are relied on:—Hoffmann's bacilli do not render acid neutral litmus glucose broth; they are not pathogenic to guinea-pigs, and do not stain with Neisser's stain. The only proof of the virulence of a type of diphtheria bacillus is that diphtheria antitoxin should be capable of neutralising in a control animal an otherwise fatal dose of the bacillus. There is no satisfactory evidence that any of the types can be converted the one into the other. Whatever type of bacillus is discovered the patient should not be isolated, unless the virulence of the bacillus is proved, or the clinical evidence supports the diagnosis of diphtheria, or there is a history of exposure to infection by this disease. In the throats of persons who have been exposed to infection, or who are living in infected institutions, true diphtheria bacilli may be found, but there is no satisfactory evidence to prove that virulent diphtheria bacilli occur in the throats of healthy persons. In scarlet fever the author found Hoffmann bacilli in the throat of 20 per cent. of the cases, and non-virulent Klebs Loeffler bacilli in 5 per cent. Twelve per cent. of scarlet-fever patients had diphtheria bacilli in the nose on admission, but these again were non-virulent. Where fibrinous rhinitis (post-scarlatinal) was present the Klebs Loeffler organism was found in all 33 cases, and in the 23 in which the inoculation test was applied these were found to be fully virulent. With regard to the origin of these cases of post-scarlatinal diphtheria, Pugh considers that the disease is not associated with any drainage defects, with overcrowding, or with the treatment of both scarlet fever and diphtheria upon the same site. Three possible factors are the increase in the number of beds in a ward beyond the normal; the introduction of unrecognised diphtheria, and the transference of infection from the throats of nurses, who although themselves healthy, are carrying diphtheria bacilli in the nose and throat after attendance upon cases of diphtheria.

[1] Berl. Klin. Woch, Aug. 25, 1902. [2] Ibid., Sept. 22, 1902. [3] Treatment, V. No. 5, p. 376. [4] Jour. Appl. Microsc IV., No. 9, 1472. [5] Ref. Med, Aug. 27, 1901. [6] Cent. f. Bakt., May 14, 1902. [7] Report Ottawa Ger., 1899. [8] Brit. Med. Jour., Nov. 25, 1902, et. seq. [9] First Ann. Rep. Asyl. Com. Metrop. Asyl. Bd., 1901.

